# Evaluation of Amphiphilic Peptide Modified Antisense Morpholino Oligonucleotides In Vitro and in Dystrophic *mdx* Mice

**DOI:** 10.3390/polym9050177

**Published:** 2017-05-15

**Authors:** Mingxing Wang, Bo Wu, Peijuan Lu, Sapana N. Shah, Jason D. Tucker, Lauren E. Bollinger, Qilong Lu

**Affiliations:** McColl-Lockwood Laboratory for Muscular Dystrophy Research, Carolinas Medical Center, 1000 Blythe Blvd, Charlotte, NC 28231, USA; Bo.Wu@carolinashealthcare.org (B.W.); Pei.Lu@carolinashealthcare.org (P.L.); Sapana.Shah@carolinashealthcare.org (S.N.S.); Jason.Tucker@carolinashealthcare.org (J.D.T.); Lauren.Bollinger@carolinashealthcare.org (L.E.B.); Qi.Lu@carolinashealthcare.org (Q.L.)

**Keywords:** amphiphilic peptide, exon-skipping, antisense oligonucleotide, muscular dystrophy

## Abstract

A series of amphiphilic peptides modified PMO (Pt-PMO) were prepared, and their antisense effect and toxicity were evaluated both in vitro and in *mdx* mice. The results showed that the exon-skipping performance of Pt-PMO are relative to the structure of the conjugated peptide: the Pt3/Pt4 composed of six/seven arginines and one myristoylation modified PMO showed more efficacy and with less toxicity as compared to others, confirming that appropriate hydrophilic-lipophilic balance (HLB) and cationic sequence numbers play a crucial role in improving cell uptake and corresponding exon-skipping efficiency. This was observed particularly in enhanced delivery efficiency of PMO comparable to B-PMO in vitro, while 6-fold improved exon-skipping was achieved against naked PMO in vivo. The multi-PMO modified Pt8-PMO also showed improved exon-skipping both in vitro and in vivo, though there is lower efficiency in systemic delivery as compared to Pt4-PMO. These data suggest that with optimization of peptide in component, charge density has clear potential for exploration towards achieving higher efficiency of antisense oligonucleotide systemic delivery, and thus is more applicable for clinical application.

## 1. Introduction

Antisense oligonucleotide (AO)-mediated exon-skipping is a promising approach for treating Duchenne muscular dystrophin (DMD). The skipping of the exon takes place on RNA level, not on DNA level, to restore the reading frame to create a truncated protein. The consequence of the treatment depends on adequate uptake of AO by the body-wide target tissues [1,2,3,4,5,6]. AOs with 2′-O-methyl-phosphorothioate RNA (2′-OMePS) and Phosphorodiamidate morpholino oligomer (PMO) chemicals have been widely deployed for exon-skipping in dystrophin transcripts and applied in clinical trials for DMD treatment [7,8,9,10,11]. Both PMO and 2′-OMePS are more resistant to RNases and DNases, improving stability in vivo. However, certain obstacles in delivery for systemic treatment must be overcome to effectively reach the nucleus of the target muscle fibers. PMO as a chemically modified DNA analogue has the deoxyribose ring replaced with morpholino ring linked through phosphorodiamidate intersubunits, making it neutral under physiological condition, therefore, exhibiting excellent stability and lower toxicity compared with other counterparts [12,13,14,15,16,17,18,19]. Recently, Sarepta Therapeutics has won US Food and Drug Administration (FDA) approval of Exondys 51 (Eteplirsen), the first drug for treating DMD. Systemic delivery of PMO has been shown to restore dystrophin protein expression in multiple peripheral muscle groups in dystrophin-deficient *mdx* mice but with low efficiencies even at high doses, due to its poor tissue uptake and rapid clearance from the bloodstream [20,21,22,23]. For this reason, significant therapeutic effect for DMD with repeated administration of oligonucleotide systemically has proven difficult in achieving its ideal clinical function. Therefore, improving cell and tissue uptake is critical for PMO to serve as an effective treatment. Cell-penetrating peptides (CPPs) can transport different cargo molecules across the plasma membrane, thus acting as molecular delivery vehicles in the treatment of different diseases [24,25,26,27]. The main characteristic of such peptides is the rich inclusion of positively charged arginine (R) or other amino acid residues. Different CPP sequence-modified PMO (PPMO), or Guanidinium functionalized Dendron-modified PMO (Vivo-PMO) have been explored, with some of them being highly effective for PMO uptake to most tissues, including the heart with significant increases in levels of exon-skipping and dystrophin restoration upon systemic treatment in *mdx* mice [14,15,16,17,19,20,21,22,23,25,26,27,28,29,30]. However, systemic toxicity including kidney tubular degeneration, blood-clotting still limits their clinical applications either [31,32]. In a recent proof-of-concept study, we have demonstrated the feasibility of cationic amphiphilic polymers enhancing exon-skipping efficiency for PMO in *mdx* mice [33,34,35]. With the purpose to maintain efficacy while reducing toxicity for PMO delivery via chemical formulation, we conjectured that flexibility of peptide’s sequences, charge density, hydrophilic–lipophilic balance (HLB) would be critical. A set of cationic amphiphilic peptides composed of arginine (R)/lysine (K) as cationic components and side-grafted myristoyl as lipophilic ingredient were designed and chemically conjugated into PMO to create peptide-PMO (Pt-PMO) macromolecules. The choice of myristic acid grafted to the peptide is due to its sufficiently high hydrophobicity to promote incorporation into the fatty acyl core of the phospholipid bilayer of the plasma membrane of the cell, for which it is widely employed in cosmetics, personal-care products, and lipophilic peptides for gene delivery [18,36]. With these principles in mind, we conducted a systematic study to identify the key molecular components responsible for transfection activity with the amphiphilic peptides, their physicochemical properties, relative toxicities, and delivery performance evaluated in vitro and in vivo in the dystrophic *mdx* mice.

## 2. Materials and Methods

Dulbecco’s Modified Eagle’s Medium (DMEM), penicillin-streptomycin, fetal bovine serum (FBS), l-glutamine and HEPES [4-(2-hydroxyethyl)-1-piperazineethanesulfonic acid] buffer solution (1 M) were purchased from GIBCO, Invitrogen Crop (Carsbad, CA, USA). Phosphorodiamidate morpholino oligomer PMOE23 (5′-GGCCAAACCTCGGCTTACCTGAAAT-3′) targeted to the murine dystrophin exon 23/intron 23 boundary site, 5′-amino PMOE23, and Endo-porter were purchased from Gene Tools (Philomath, OR, USA). The designed amphiphilic peptides were customer ordered from Genescript (Piscataway, NJ, USA). All other chemicals were purchased from Sigma-Aldrich (St. Louis, MO, USA), unless otherwise stated.

### 2.1. Synthesis and Characterization of Peptide-PMO Conjugates

The 5-amino modified PMO E23 was firstly activated with *N*-[γ-maleimidobutyryloxy] succinimide ester (GMBS, Sigma-Aldrich, St. Louis, MO, USA) linker, and then reacted with cysteine-modified peptides as literature reported with some modification (Scheme 1 in Supplementary Materials) [14,15,16,17,19,25,26,27]. Briefly, excess of the cross-linker GMBS was added to the 5′-amine modified PMO (1000 nmol) solution in 50 mM sodium phosophate buffer (pH 7.2) containing 20% acetonitrile at room temperature (r.t.). The mixture was stirred at this temperature for 2 h. The intermediate GMBS-linked PMO was precipitated using cold acetone and dried in vacuum. A two-fold molar excess of the peptide was added to the above GMBS-linked PMO in 50 mM sodium phosphate buffer (pH 6.5) containing 20% acetonitrile and allowed to incubate at r.t. in the dark for 4 h. The product was purified by gel filtration using a column with Sephadex G-100 gel (GE Healthcare), Amicon centrifuge MWCO 3000 to remove the unconjugated peptides or other small molecules. The samples were then lyophilized and characterized by reversed-phase HPLC and by MALDI-TOF mass spectrometry (Figures S1 and S2 in Supplementary Materials). The code and sequences of peptides and corresponding peptide-PMO conjugates are listed in Table 1. 

### 2.2. Cytotoxicity in C2C12E23 Cell Line

Cytotoxicity was evaluated in C2C12E23 cell line using the MTS [3-(4,5-dimethylthiazol-2-yl)-5-(3-carboxymethoxyphenyl)-2-(4-sulfophenyl)-2H-tetrazolium]-based assay. Briefly, the cells were seeded in a 96-well tissue culture plate at 1 × 10^4^ cells/well in 200 µL medium containing 10% FBS, then exposed to polymers at different doses once it achieves 70–80% confluence, followed by 24 h incubation at 37 °C and 10% CO_2_. Twenty (20) µL of Cell Titer 96 Aqueous One Solution Reagent was added to each well, and further incubation for 4 h. The absorbance was measured at 570 nm using Tecan 500 Plate reader (Tecan US Inc., Morrisville, NC, USA) to obtain the metabolic activity of the cell. Viability of untreated cells was taken as 100% and wells without cells were used as blanks. The relative cell viability was calculated by: (*A*_treated_ − *A*_background_) × 100/(*A*_control_ − *A*_background_). All viability assays were carried out in triplicate [33,34,35]. 

### 2.3. Exon-Skipping In Vitro 

The C2C12E23 cell line expressing the reporter GFP was used in this study. The expression of GFP in the cells was controlled by the effective skipping of the inserted mouse dystrophin exon 23 (mDysE23) within the GFP coding sequence. C2C12E23 cells were grown in DMEM and maintained at 37 °C and 10% CO_2_ in a humidified incubator. Then, 5 × 10^4^ cells/well were seeded in a 24-well plate in 500 µL medium containing 10% FBS and grown to reach 70–80% confluence prior to transfection. Cell culture medium was replaced prior to addition of Pt-PMO (fixed at 5 µg), while B-PMO and PMO were used as controls. Transfection efficiencies were visualized after 72 h incubation with the Olympus IX71 fluorescent microscope (Olympus America Inc., Melville, NY, USA) and digital images taken with the DP Controller and DP Manager software. The exon-skipping was further examined by reverse transcriptase (RT)-PCR [20,21,22,23]: Collected cells were initially washed twice with phosphate-buffered saline (PBS), and RNA was extracted as follows: Cells were treated with 1 mL TRIzol reagent (Invitrogene, Carlsbad, CA, USA) for lysis, kept 5 min’ incubation at r.t., then to the mixtures were added 0.2 mL of chloroform, and the samples were shaken vigorously for 15 s, incubated 5 min at r.t., and centrifuged for 15 min at 12,000× *g* at 4 °C. The aqueous phase was transferred to a fresh tube, and added 0.5 mL of isopropyl alcohol, and incubated samples at r.t. for 10 min, then centrifuged at 12,000× *g* for 15 min at 4 °C. The supernatants were removed and the RNA pellets were washed once with 1 mL 75% ethanol. The sample tubes were gently shaken and centrifuged at 7500× *g* for 5 min at 2–8 °C. Then RNA pellets were air-dried for 10 min, and dissolved RNA in RNase-free water, and incubated for 10 min at 55 °C. Finally, the RNA was stored at −80 °C for later use. RT-PCR was performed with RT-Fidelitaq MasterMix (USB, Cleveland, OH, USA) to amplify the sequence of interest. 100 ng of template RNA was used for each 25 µL RT-PCR reaction. The primer sequences for the RT-PCR were eGFP5′, 5′-CAGAATTCTGCCAATTGCTGAG-3′ and eGFP3′, 5′-TTCTTCAGCTTGTGTCATCC-3′. The cycle conditions for reverse transcription were 43 °C for15 min, 94 °C for 2 min. The reaction was then cycled 30 times at 94 °C for 30 s, 65 °C for 30 s, and 68 °C for 1 min. The products were examined by electrophoresis on a 1.5% agarose gel. The intensity of the bands was measured with the National Institutes of Health (NIH) ImageJ 1.42 and percentage of exon-skipping was calculated with the intensity of the two bands representing both exon 23 unskipped and skipped as 100%. Unskipped band including exon 23 (upband) is 424 bp, skipped band without exon 23 is 211 bp.

### 2.4. In Vivo AO Delivery and Immunohistochemistry

This study was carried out in strict accordance with the recommendations in the Guide for the Care and Use of Laboratory Animals of the National Institutes of Health. The protocols were approved by the Institutional Animal Care and Use Committee (IACUC), Carolinas Medical Center (Breeding protocol: 10-13-07A; Experimental protocol: 10-13-08A). All injections were performed under isoflurane anesthesia, and all efforts were made to minimize suffering [20,21,22,23,33,34,35]. 

#### 2.4.1. Animals and Injections

Dystrophic *mdx* mice aged 4–5 weeks were used for in vivo testing (five mice each in the test and control groups) unless otherwise stated. Mice were killed by CO_2_ inhalation at desired time points, and muscles and other issues were snap-frozen in liquid nitrogen-cooled isopentane and stored at −80 °C. The PMOE23 (Gene Tools, Philomath, OR, USA) was used. For intramuscular (i.m.) injections, 2 µg Pt-PMO or PMO only in 40 µL saline was applied for each tibialis anterior (TA) muscle. For intravenous (i.v.) injection, 1 mg Pt-PMO or PMO only in 100 µL saline used. The muscles were examined two weeks later. 

#### 2.4.2. Immunohistochemistry and Histology

Serial sections of 6 µm were cut from the treated mice muscles. The sections were stained with a rabbit polyclonal antibody P7 for the detection of dystrophin protein as described previously [20,21,22,23]. Polyclonal antibodies were detected by goat anti-rabbit IgG Alexa 594 (Invitrogen, Carlsbad, CA, USA). As for dystrophin-positive fiber counting, the number of dystrophin-positive fibers in one section was addressed using the Olympus BX51 fluorescent microscope (Olympus America Inc., Melville, NY, USA), and the muscle fibers were defined as dystrophin-positive when more than two-thirds of a fiber membrane showed continuous staining.

#### 2.4.3. Western Blot and RT-PCR for In Vivo Samples

Protein extraction and Western blot were done as described previously [20,21,22,23]. The collected sections were ground into powder, and lysed with 200 µL protein extraction buffer [1% Triton X-100 (Sigma-Aldrich, St. Louis, MO, USA), 50 mM Tris pH 8.0, 150 mM NaCl, 0.1% sodium dodecyl sulfate (SDS)], boiled at 100 °C water for 1 min, then centrifuged at 18,000× *g* at 4 °C for 15 min. The Supernatants were quantified for the protein concentration with a protein assay kit (Bio-Rad Laboratories, Hercules, CA, USA). Proteins were loaded onto a 4% to 15% Tris-HCL gradient gel. Samples were electrophoresed 4 h at 120 V at r.t. Then, the gel blotted onto nitrocellulose membrane for 4 h at 150 V at 4 °C. The membrane was probed with NCL-DYS1 monoclonal antibody against dystrophin rod domain (1:200 dilutions, Vector Laboratories, Burlingame, CA, USA). The bound primary antibody was detected by HRP-conjugated goat anti-mouse IgG (1:3000 dilutions, Santa Cruz Biotechnology, Santa Cruz, CA, USA) and the ECL Western Blotting Analysis System (Perkin-Elmer, Waltham, MA, USA). The intensity of the bands of products obtained from the treated *mdx* mice muscles was measured, and is compared with that of normal muscles of C57BL6 mice as 100% (National Institutes of Health, ImageJ software 1.42). α-Actin was detected by rabbit anti-actin antibody (Sigma, St. Louis, MO, USA) as a sample loading control. 

Total RNA was extracted from the muscle after dissection, 100 ng of RNA template was used for a 25 µL RT-PCR with the Fidelitaq RT-MasterMix (USB, Cleveland, OH, USA). The primer sequences for the RT-PCR were Ex20Fo 5′-CAGAATTCTGCCAATTGCTGAG-3’ and Ex26Ro 5′-TTCTTCAGCTTGTGTCATCC-3′ for amplification of mRNA from exons 20–26. Unskipped band including exon 23 (upband) is 1093 bp, skipped band without exon 23 is 880 bp. 

### 2.5. Measurement of Serum Creatine Kinase and Other Components

Mouse blood was taken immediately after cervical dislocation and centrifuged at 1500 r.p.m. for 10 min. Serum was separated and stored at −80 °C. The level of serum components was determined by IDEXX Laboratories (North Grafton, MA, USA). 

### 2.6. Transmission Electron Microscopy (TEM)

The Pt-PMO or Pt/PMO polyplex solution containing 2 µg of PMO was prepared in 0.2 mL medium, analyzed using Transmission Electron Microscopy (TEM, Philips CM-10, Philips Electronic North America Corp., Andover, MA, USA). The samples were prepared using negative staining with 1% phosphotungstic acid as reported [33,34,35]. Briefly, one drop of sample solution was placed on a formvar and carbon coated carbon grid (Electron Microscopy Sciences, Hatfield, PA, USA) for 1 h, and the grid was blotted dry, followed by staining for 3 min. The grids were blotted dry again. Samples were analyzed at 60 kV. Digital images were captured with a digital camera system from 4 pi Analysis (Durham, NC, USA). 

### 2.7. Statistical Analysis

The statistical analysis of experimental data was evaluated using both one-way analysis of variance (ANOVA) and Two-tailed Student’s *t*-test, and results were reported as mean values ± SEM. Statistical significance was accepted when * *p* ≤ 0.05. 

## 3. Results and Discussion 

### 3.1. Cytotoxicity

**Figure 1:**
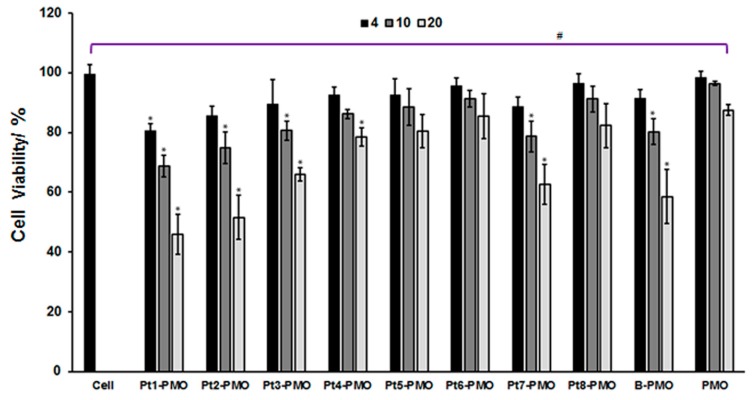
Cytotoxicity of peptide-PMO conjugates at different dosages determined by MTS assay in C2C12E23 cell line at the concentration of 4, 10, 20 µg/mL (the cells were plated in 96-well plate at an initial density of 1 × 10^4^ cells/well in 0.1 mL of growth medium). The results are presented as the mean ± SEM (one-way analysis of variance test, # *p* ≤ 0.05 indicates a significant difference between groups; Two-tailed Student’s *t*-test, * *p* ≤ 0.05 compared with untreated cells, *n* = 3).

The C2C12 myoblast with mouse bifurcated dystrophin exon 23 inserted (C2C12E23) cultured to differentiating myotubes was used for in vitro study. Restoration of GFP expression relies on the targeted removal of exon 23 through AO-induced exon-skipping. Firstly, the cytotoxicity of the peptide-PMO conjugates at different doses (4, 10, 20 µg/mL), were assessed in the C2C12E23 cell line via the MTS [3-(4,5-dimethylthiazol-2-yl)-5-(3-carboxymethoxyphenyl)-2-(4-sulfophenyl)-2H-tetrazolium] viability assay as shown in Figure 1. The results indicate that the more Rs contained within the peptide, the more toxic it is, such as the toxicity of peptides is in the order: Pt3-PMO (7 Rs) > Pt4-PMO (6 Rs) > Pt5-PMO (5 Rs) owning one myristoylation; and Pt1-PMO (8 Rs) > Pt2-PMO (7 Rs) with two myristoylation; Pt1/Pt2-PMOs showed more toxicity than others having one myristoylation; Pt7-PMO without R displayed less toxic than Pt1/Pt2-PMO, but more toxic compared with Pt3/Pt4/Pt5/Pt6-PMO, probably due to its two myristoylation. Arg-Gly-Asp (RGD) motif-based Pt6-PMO owing 4 Rs without myristoylation is the least toxic among them, the cell viability goes up 85%, and Pt4/Pt5/Pt8-PMO also reached around 80% cell viability even at dose up to 20 µg/mL. Pt8-PMO is the molecule that four PMO were conjugated into one peptide unit, making it less peptide ingredient within the whole molecule as compared to others, the macromolecular conjugate is capable to convey multiple oligonucleotides in a single uptake, having less toxic potential. Pt1/Pt2-PMO showed slightly more toxicity than B-PMO (B-peptide conjugated PMO produced by AVI BioPharma), the alive cell fells to 45%, 51% at the high dose of 20 µg/mL, respectively; while, Pt7-PMO with two myristoylations showed relatively higher cell viability compared with Pt1/Pt2-PMO containing the same two myristoylations, likely due to the absence of R. These data further demonstrated that the more positive charge and/or the more hydrophobic a peptide, the more associated toxicity it has [25,26,27]. The introduction of one myristoylation did not cause much toxicity, otherwise probably playing an essential role in membrane targeting, and improving cell-uptake [37]. 

### 3.2. Delivery In Vitro

**Figure 2:**
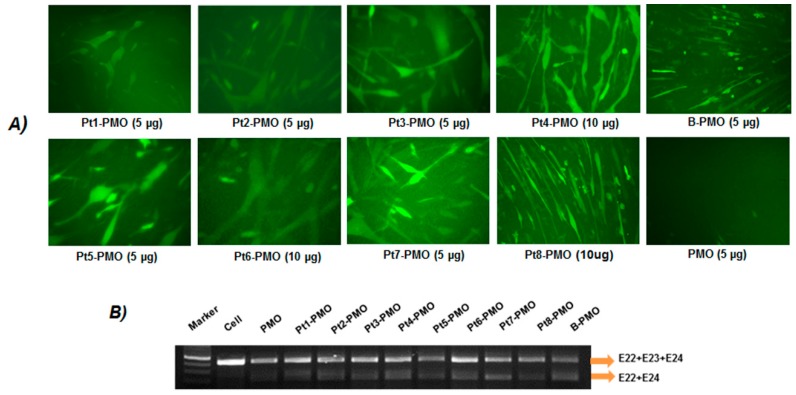
GFP expression induced by Pt-PMO (5, 10 µg) in C2C12E23 cells and B-PMO (5 µg) as control in 0.5 mL 10% FBS-DMEM after 72 h treatment. (**A**) Fluorescence detection for GFP expression, original magnification: 200×; (**B**) RT-PCR of exon 23 skipped Pt-PMO at the dose of 5 µg.

The C2C12E23 reporter cell uses a muscle creatine kinase (MCK) promoter to drive the expression of GFP, thus allowing us to evaluate the exon-skipping of antisense oligonucleotides in differentiating or differentiated cells. Firstly, the cells were treated with Pt-PMOE23 at four doses (2, 5, 10, 20 µg in 0.5 mL 10% FBS-DMEM) and the delivery performances were visualized by fluorescence microscopy. As predicted, Pt-PMOs enhanced exon-skipping compared to PMO alone even at the lowest dose of 2 µg, and dose-dependent GFP expression was observed with the most effective dosage around 5 to 10 µg. No apparent GFP enhancement was further obtained when the dose of Pt-PMO increased to 20 µg, probably caused by aggregation of self-assemble at high concentration, which was exemplified by Pt4-PMO (Figure S3 in Supplementary Materials). We then focused on the evaluation of exon-skipping of the conjugates at the two doses (5, 10 µg), with GFP expression visualized by microscopy, and quantitatively analyzing the levels of exon-skipping by RT-PCR. The detection of exon 23 skipping was 13.7%, 32.5%, 43.9%, 52.5%, 60.5%, 49.7%, 39.8%, 58.7%, 56.8% and 62.5% for PMO, Pt1-PMO, Pt2-PMO, Pt3-PMO, Pt4-PMO, Pt5-PMO, Pt6-PMO, Pt7-PMO, Pt8-PMO and B-PMO under the dose of 5 µg, respectively. Especially the Pt3-PMO, Pt4-PMO and Pt5-PMO with one myristoylation and six/seven Rs, Pt-7 with two myristoylations and without R showed greater efficacy than others, the multi-PMO conjugates Pt8-PMO without myristolytion also showed more effective at the optimal dose, probably resulted from its larger size making higher drug-loading efficiency. Further, no additional benefits were observed from RGD-containing Pt5/Pt6-PMOs, even the RGD in dendritic modification (Pt6), implying that the RGD motif-based targeting effect is limited. The efficiency in exon-skipping might be mainly related to the charges and HLB of the peptides. This is indicated by much higher exon-skipping efficiency obtained with Pt3-PMO, Pt4-PMO or Pt5-PMO containing a moderate number of *R* and single myristoylation, the Pt7-PMO without R showing higher efficiency probably caused by its more hydrophobicity as compared to other counterparts [33,34,35]. Conversely, the Pt1/Pt2-PMO produced lower delivery efficiency against others tested, as it demonstrates greater toxicity resulted from the more cationic peptide sequences and two myristoylation inclusions. In general, the amphiphilic peptide constructing a moderate number of *R* and single myristoylation modified PMOs showed improved delivery activity over unmodified PMO in vitro (Figure 2). 

### 3.3. Delivery In Vivo 

#### 3.3.1. Local Delivery

**Figure 3:**
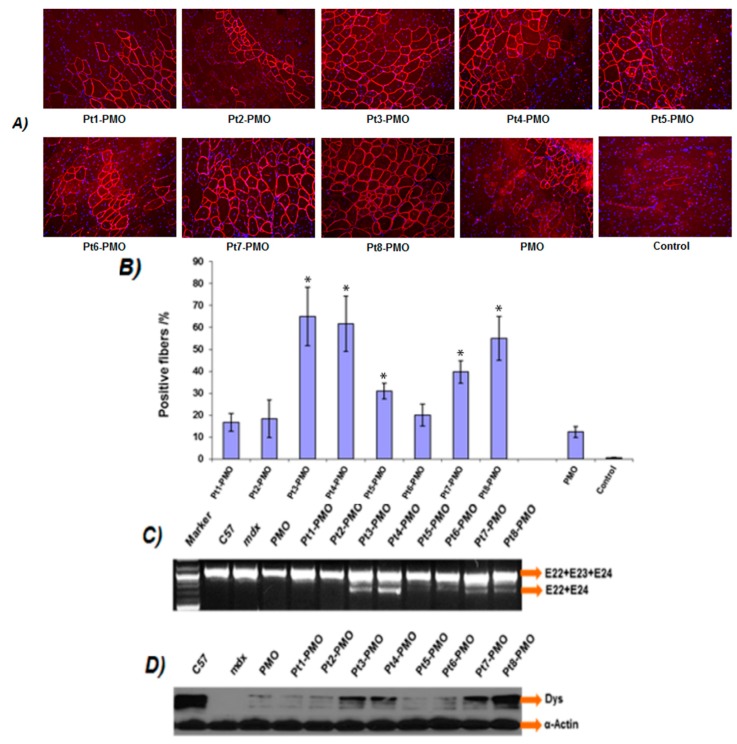
Dystrophin exon-skipping and protein expression following intramuscular (i.m.) administration of PMOE23 in Tibialis anterior (TA) muscles of *mdx* mice (aged 4–5 weeks) after two-week treatment. Muscles were treated with Pt-PMO or PMO (2 µg) in 40 µL saline: (**A**) restoration of dystrophin in TA muscles was detected by immunohistochemistry using rabbit polyclonal antibody P7 against dystrophin. Blue nuclear staining with DAPI (4,6-diamidino-2-phenylindole), original magnification: 200×; (**B**) The percentage of dystrophin-positive fibers. The numbers of dystrophin-positive fibers were counted in a single cross-section (*n* = 5, Two-tailed Student *t*-test, * *p* ≤ 0.05 compared with 2 µg PMO); (**C**) Detection of exon 23 skipping by reverse transcriptase PCR (RT-PCR) and sequence confirmation. Total RNA of 100 ng from each sample was used for amplification of dystrophin mRNA from exon 20 to exon 26. The upper bands (indicated by E22 + E23 + E24) correspond to the normal mRNA, and the lower bands (indicated by E22 + E24) correspond to the truncated mRNA with exon E23 skipped; (**D**) Western blot demonstrating the expression of dystrophin protein (50% protein of WT C57 used compared with PMO-treated mice). Dys, dystrophin detected with monoclonal antibody Dys 1. α-Actin was used as the loading control.

The effect of the Pt-PMO in vivo was firstly evaluated by intramuscular (i.m.) injection in tibialis anterior (TA) muscles of *mdx* mice (aged 4–5 weeks). The *mdx* mouse contains a nonsense mutation in dystrophin exon 23 preventing production of a functional dystrophin protein, whereas targeted removal of the mutated exon through exon-skipping can restore the reading frame and expression of dystrophin protein. Each TA muscle was treated with 2 µg Pt-PMO or PMO in 40 µL saline, and the treated muscles were harvested two weeks after injection. Immunohistochemistry showed that PMO alone induced around 10% dystrophin positive fibers in one cross-section of the TA muscle, whereas, dramatically increased dystrophin positive fibers were observed in the TA muscles treated with Pt-PMOs, reaching 65%, 62% and 56% for Pt3-PMO, Pt4-PMO and Pt8-PMO, respectively (Figure 3). The levels of exon-skipping and dystrophin expression were further confirmed by RT-PCR and Western-blot studies. Consistent with low efficiency in cell culture, Pt1/Pt2-PMOs produced low dystrophin expression likely due to their higher cationic leading to more nonspecific targeting, though the mechanism remains unclear; while the Pt5/Pt6-PMO showed lower efficiency probably owing to its fewer number of R or absence of myristoylation. The Pt3/Pt4 modified PMOs produced enhanced exon-skipping, likely resulted from their optimized sequences and HLB; the Pt8-PMO showed higher efficiency, probably benefited from its larger molecule size and high drug-loading efficiency compared with others (peptide-PMO equimolar conjugates). Histological examination by Hematoxylin and Eosin (H & E) (Burlingame, CA, USA) staining showed no signs of elevated inflammation or fiber degeneration and regeneration as compared to the saline-treated TA muscles, reconfirming their low tissue toxicity at the tested dosages via local injection.

#### 3.3.2. Systemic Delivery

**Figure 4:**
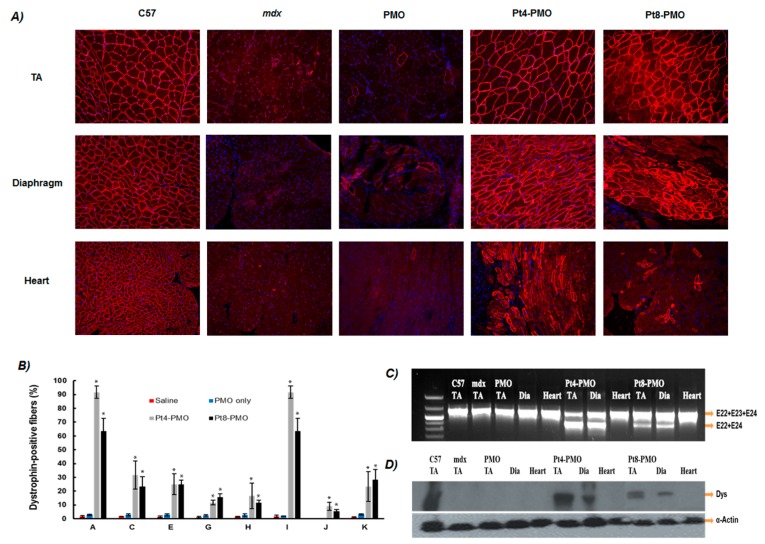
Restoration of dystrophin expression after systemic delivery of Pt-PMOs in *mdx* mice (aged 4–5 weeks). Each mouse was injected with 1.0 mg Pt-PMO or PMO: (**A**) Restoration of dystrophin expression after two weeks intravenous (i.v.) administration in tibialis anterior (TA), diaphragm and heart muscles, original magnification: 200×; (**B**) The quantification of dystrophin positive fibers expressed as percentage levels based on the positive fibers covered area within one section from TA, diaphragm and the heart muscle of the treatment groups (*n* = 5, Two-tailed Student *t*-test, * *p* ≤ 0.05 compared with PMO only); (**C**) Detection of dystrophin exon 23 skipping by RT-PCR. E22 + E23 + E24 and E22 + E24 representing normal mRNA and the mRNA with exon 23 skipped, respectively; (**D**) Western blot demonstrating the levels of dystrophin expression in the TA muscle, diaphragm and the heart, α-actin used as a loading control. Note: A: Tibialis anterior; C: Quadriceps; E: Gastrocnemius; G: Abdomen; H: Intercostals; I: Diaphragm; J: Heart; K: Bicep.

DMD is a systemic disease, affecting body-wide muscles including cardiac muscle, therefore systemic treatment is essential. Considering together the results in in vitro and in vivo local administration, we explored the two effective conjugates (Pt4-PMO and Pt8-PMO) for their potential systemic effect by intravenous (i.v.) injection at the dose of 1.0 mg/mouse. Immunohistochemistry demonstrated that Pt-PMOs produced ≥15% dystrophin positive fibers in all skeletal muscles, being over 90%, 65% positive fibers observed in TA and diaphragm from *mdx* mice treated with the single dose of 1 mg Pt-PMO, respectively; and, more importantly, membrane-localized dystrophin about 10%, 5% of cardiac muscle fibers in some areas of the heart obtained with Pt4-PMO, Pt8-PMO, correspondingly. In contrast, as control 1 mg PMO induced dystrophin expression in less than 3% of muscle fibers in all skeletal muscles and no detectable dystrophin in cardiac muscle when examined two weeks after injection (Figure 4). The clear detection of dystrophin induction in cardiac muscle could be crucial even at low levels, since cardiomyopathies due to lack of dystrophin expression is currently the leading cause of death among DMD patients [14,15,16,17,18,19]. In addition, a more effective restoration of dystrophin in skeletal muscles may in turn exacerbate the failure of heart function if dystrophin expression cannot be effectively restored in cardiac muscle. 

Consistent with the immunohistochemistry, RT-PCR results showed that TA and diaphragm muscles with Pt4-PMO-treated mice contained spliced form of dystrophin mRNA with exon 23 skipped as the major species, being above 70% of the total product; while Pt8-PMO achieved around 30% exon 23 skipping, probably due to the low peptide ingredient within the conjugate and reduced tissue-uptake as compared to the Pt4-PMO. Expression levels of dystrophin protein in TA and diaphragm muscles measured by Western blots for Pt4-PMO-treated mice were over 70%, 40% of the levels of the normal control muscle obtained, respectively. It is difficult to detect in the heart caused by low level (only 7–13%) of dystrophin positive fibers observed. Pt8-PMO-treated ones were lower as compared to Pt4-PMO administrated, which is in line with the exon-skipping outcome of RT-PCR. 

**Figure 5:**
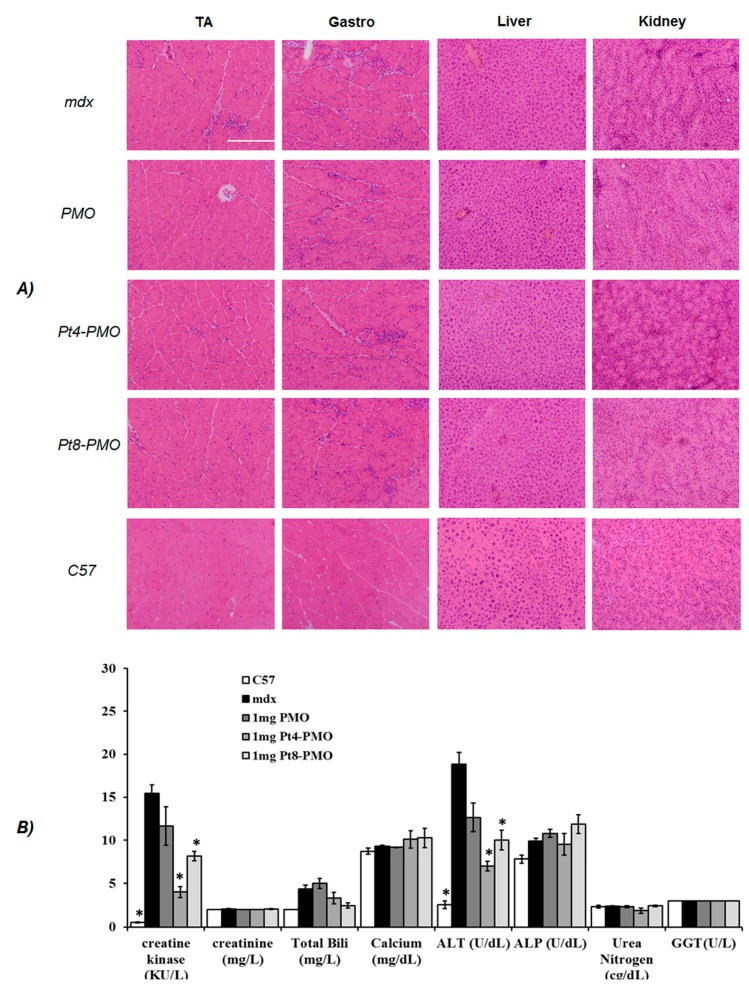
Examination of pathology and serum tested after PMO treatment: (**A**) Hematoxylin and Eosin (H & E) staining of tibialis anterior, gastrocnemius muscles, liver and kidney tissue of Pt-PMO delivery from the normal C57BL6 mice (C57), untreated *mdx* mice, PMO- and Pt-PMO-treated *mdx* mice, scale bar = 200 µm; (**B**) Serum testing. CK, creatine kinase ((kU/L), creatinine (mg/L), urea nitrogen (cg/dL), total bilirubin (mg/L), Calcium (mg/dL), anlanie transaminase (ALT) (U/dL), alkaline phosphatase (ALP) (U/dL) and gamma-glutamyltransferase (GGT) (U/L) (*n* = 5, Two-tailed Student *t*-test, * *p* ≤ 0.05).

During treatment with these Pt-PMO conjugates, we observed no signs of abnormal behavior or change in body weight and overall condition under systemic delivery. Histological examination revealed no pathological changes in skeletal muscles, liver, kidney and lung compared to untreated *mdx* mice by H & E staining (Figure 5A). Significant reduction in serum creatine kinase levels was observed in the Pt-PMO-treated mice compared with PMO-alone-treated *mdx* mice, whereas urea nitrogen, total bilirubin, direct bilirubin, alkaline phosphatase, and γ-glutamyltransferase were normal (Figure 5B). These results, together with the lack of any clearly detectable toxicity in muscles after systemic treatment, further solidify that the modification of PMO with optimized peptides has the potential to increase exon-skipping especially for the treatment of muscular dystrophies including long-term administration, though it has not been the ideal structure leading to expected therapeutic value. 

### 3.4. Morphology Study by Transmission Electron Microscopy (TEM)

**Figure 6:**
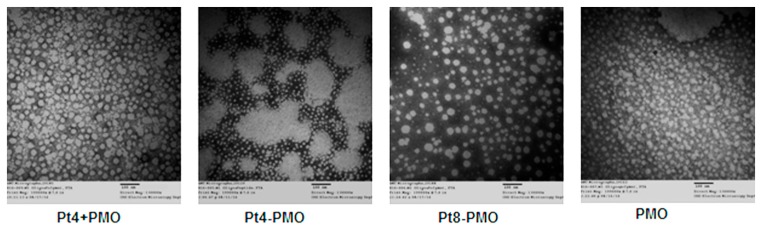
Negatively stained transmission electron micrographs (scale bar = 100 nm; Direct Mag: 130,000×).

We hypothesized that peptide conjugation would improve the delivery efficiency due to homogeneity of nanoparticle distribution compared with the conventional peptide/PMO formulation. To specifically test this, the Pt4-PMO, Pt8-PMO, Pt4 + PMO (Pt4 mixed PMO) and PMO alone morphology were visualized under TEM. The affinity between peptide and oligonucleotides is important to affect their delivery into cell or tissue, because the promoting delivery of oligonucleotides is to incorporate it into nano-sized particles, which helps to overcome innate biological barriers, increase uptake, and enhance escape from membrane compartments for effect once internalized. As illustrated in Figure 6, the Pt4-PMO conjugate formed very uniform particles size below 20 nm due to its single molecule system and ordered molecular arrangement. Whereas, the Pt4 mixed with PMO (1:1 composition) resulted in increased particles sized from 30 to 60 nm, this nanoparticle formation is due to the amphipathicity of the complex from the relatively hydrophobic PMO portion and a relatively hydrophilic peptide part, a structure susceptible to form micelle, which is consistent with that reported previously [38]. The Pt8-PMO conjugate produced particles with a different distribution and size around 20–70 nm, most likely due to its multi-PMO conjugate within one peptide making its more steric hindrance and repulsive force between the conjugates compared with Pt4-PMO conjugate with a 1:1 stoichiometry. Whereas, the PMO oligonucleotides alone formed particles of different sizes below 50 nm, likely a result of hydrophobic interactions among PMO molecules. Overall, the Pt4-PMO conjugate produced well-defined particles, which may lead to a higher tolerance level of serum and more effective delivery of PMO into the vicinity of muscles and improve the uptake of PMO through the vasculature and tissue membranes. 

## 4. Conclusions

In summary, a series of cationic amphiphilic peptide modified PMOs has been successfully prepared and valuated for exon-skipping in vitro and in dystrophic *mdx* mice. The results upon the structure-delivery performance relationship demonstrated that Pt3/Pt4 consisting of six or seven Rs and one myristoylation are more effective, and less toxic compared with others, indicating that the appropriate HLB and the numbers of cationic R play key roles in achieving uptake and exon-skipping efficiency. The most effective Pt4-PMO provides exon-skipping efficiency of PMO comparable to B-PMO in vitro, and 6-fold increased as compared with PMO alone in vivo. On the other hand, the multi-PMO modified Pt8-PMO macromolecule conducted better exon-skipping of PMO both in vitro and in vivo, though there is lower efficiency in systemic delivery against Pt4-PMO, which confirm the cationic amphiphilic peptide′s contribution to the delivery program of PMO, nonetheless the mechanism remains to be further clarified. These results indicated clearly that the peptide with at least six Rs and one myristoylation exhibited great activity, while with over eight Rs or two myristoylation exhibited high toxicity. These data suggest that optimization of peptide component, HLB and charge distribution can achieve enhanced exon-skipping of AO. Cationic amphiphilic peptide conjugated PMO are an encouraging potential strategy to enhance exon-skipping of AO through rational structure design to achieve improved efficacy with tolerable toxicity. 

## Figures and Tables

**Table 1:** Applied peptide sequences and code of Peptide-PMO conjugates.

Code of peptide-PMO	Peptides	Peptide sequence	Numbers of arginine	Numbers of myristoyl
Pt1-PMO	Pt1	RXRRBRK(Myr)BRK(Myr)RXRRB	8	2
Pt2-PMO	Pt2	RXRRBRK(Myr)K(Myr)RXRRB	7	2
Pt3-PMO	Pt3	RXRRBRK(Myr)RRBRXB	7	1
Pt4-PMO	Pt4	RBRRK(Myr)RRBRXB	6	1
Pt5-PMO	Pt5	RGDRK(Myr)RRBRXB	5	1
Pt6-PMO	Pt6	[(RGD)_2_K]_2_K-X	4	0
Pt7-PMO	Pt7	CKWKS(Myr)S(Myr)	0	2
Pt8-PMO	Pt8	RXRRBRRXRRBRXE(Cys)BE(Cys)BE(Cys)B	8	0
B-PMO	B	RXRRBRRXRRBRXB	8	0

X: 6-Amino hexanoic acid; B: β-alanine; Myr: Myristic acid; Cys: Cysteine; PMOE23 Sequence: GGCCAAACCTCGGCTTACCTGAAAT. PMO = Phosphorodiamidate morpholino oligomer.

## Supplementary Materials

The following are available online at www.mdpi.com/2073-4360/9/5/177/s1, Scheme 1: Peptide-PMO conjugates, Figure S1: HPLC of Pt3-PMO, Figure S2: MALDI-TOF Mass of Pt3-PMO conjugate, Figure S3: Dose-dependent PMO delivery in C2C12E23 cells. Peptide modified PMOs were used at the doses of 2, 5, 10, 20 µg in 500 µL 10% FBS-DMEM, original magnification: 200×.
