# Supplementary material for: Evaluation of Amphiphilic Peptide Modified Antisense Morpholino Oligonucleotides In Vitro and in Dystrophic mdx Mice
Source: Polymers (Basel). 2017 May 15;9(5):177. doi: 10.3390/polym9050177 (PMC6432210; doi:10.3390/polym9050177)
Supplement: Supplementary file 1 [file polymers-09-00177-s001.pdf]

Supplementary Materials: Evaluation of Amphiphilic Peptide Modified Antisense Morpholino Oligonucleotides In Vitro and in Dystrophic *mdx* Mice

Mingxing Wang, Bo Wu, Peijuan Lu, Sapana N. Shah, Jason D. Tucker, Lauren E. Bollinger and Qilong Lu

The Peptide-PMO conjugates were prepared as following synthetic scheme.

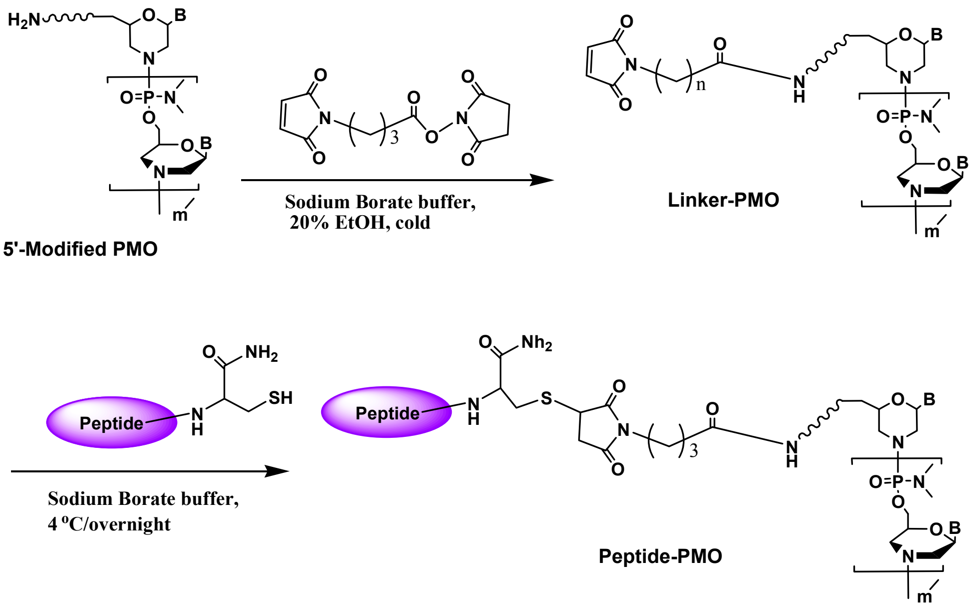

Scheme 1. Peptide-PMO conjugates.

The final products were characterized by reversed-phase HPLC (Jupiter C18, 250 mm × 4.6 mm, 5 micron) with buffer A, 0.1% trifluoroacetic acid (TFA) in water, and buffer B, 90% acetonitrile in 0.1% TFA (gradient: 40%–50% B in 25 min) with detection at 260 nm (Figure S1) and by MALDI-TOF mass spectrometry (Figure S2, on an Applied Biosystems Voyager DE-PRO (Foster City, CA, USA) using a matrix of 2,6-dihydroxyacetophenone (20 mg/mL) in the presence of 40 mg diammonium hydrogen citrate dissolved in methanol/water (1:1, *v*/*v*).

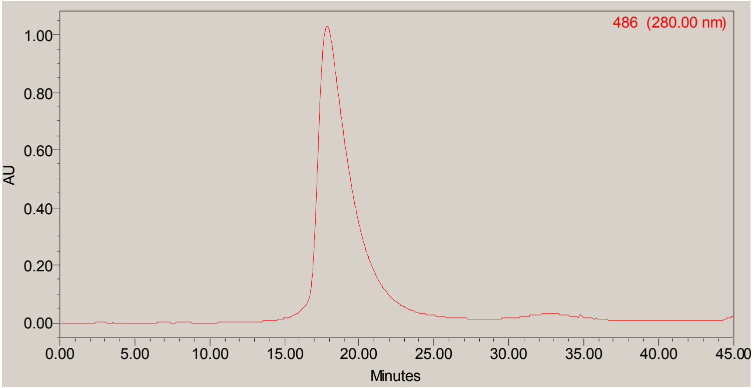

Figure S1. HPLC of Pt3-PMO.

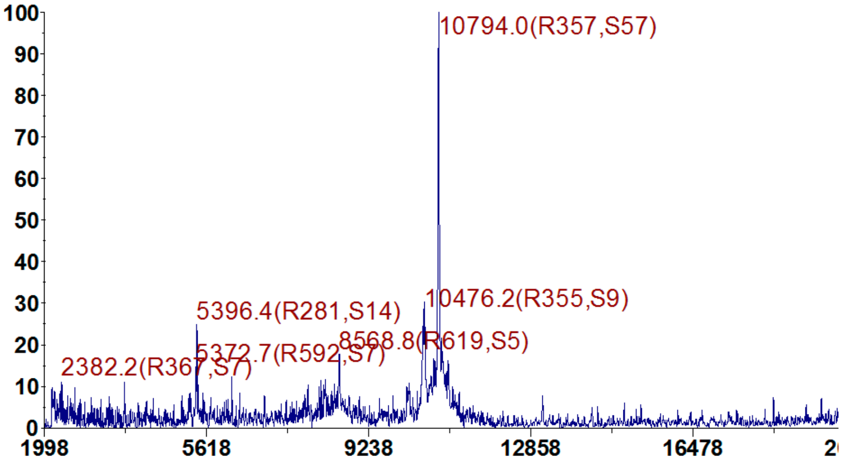

Figure S2. MALDI-TOF Mass of Pt3-PMO conjugate.

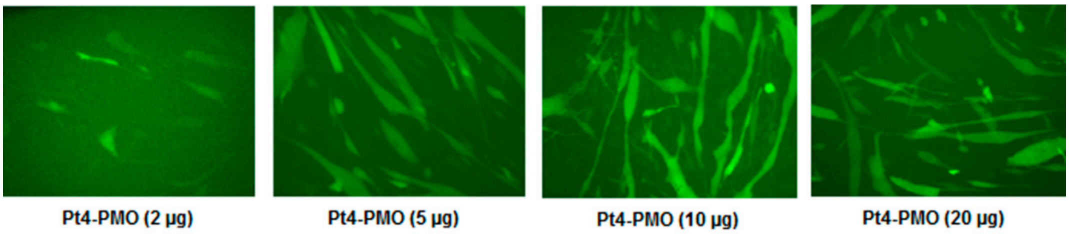

Figure S3. Dose-dependent PMO delivery in C2C12E23 cells. Peptide-modified PMOs were used at the doses of 2, 5, 10, 20 µg in 500 µL 10% FBS-DMEM, original magnification: 200×.
